# The grazing gait, and implications of toppling table geometry for primate footfall sequences

**DOI:** 10.1098/rsbl.2018.0137

**Published:** 2018-05-16

**Authors:** James R. Usherwood, Benjamin J. H. Smith

**Affiliations:** Structure and Motion Laboratory, The Royal Veterinary College, North Mymms, Hatfield, Herts AL9 7TA, UK

**Keywords:** gait, walk, static stability, quadruped

## Abstract

Many medium and large herbivores locomote forwards very slowly and intermittently when grazing. While the footfall order during grazing is the same as for walking, the relative fore–hind timing—phasing—is quite different. Extended periods of static stability are clearly required during grazing; however, stability requirements are insufficient to account for the timing. Aspects of relatively rapid rolling and pitching—toppling due to the resistance of the back to bending and twisting—can be included in a simplifying geometric model to explain the observation that, in grazing livestock, a step forward with a forefoot is consistently and immediately followed by a step forward from the hind; but not vice versa. The same principles and geometry, but applied to the footfall pattern of walking primates, show that toppling would occur at a different point in the gait cycle. This provides a potential account for the distinctive diagonal-sequence footfall pattern of primates, as it prevents the instant of toppling from being at forefoot placement. Careful and controlled hand positioning would thus be facilitated, presumably beneficial to walking on top of branches, despite a slight energetic cost compared with the usual lateral sequence pattern of horses.

## 1. Background

Attempts to account for the gaits of slow quadrupeds extend back to at least Aristotle (350 BCE, here as translated by A.S.L. Farquharson [1, §14]):

… the hind limbs move criss-cross with the fore limbs; after the off fore they move the near hind, then the near fore, and then the off hind. The reason is that (a) if they moved the forelegs together and first … *it is hard to make a continuous change of place* [i] … (b) *if they moved both the right legs first the weight would be outside the supporting limbs and they would fall* [ii]. …

This account for the normal walking quadrupedal gait exemplified by the horse clearly identifies both issues related to mechanical work [i] and static stability [ii]. Perhaps remarkably, the relative importance of each of these factors remains poorly understood. The conditions for static stability—maintaining the centre of mass position directly above some polygon of support formed by the placement of the feet (see electronic supplementary material, movie S1)—have been described graphically [2] and mathematically [3], showing the requirement of the lateral sequence footfall pattern (starting the cycle with a hindlimb, so Hind Left (HL), Fore Left (FL), Hind Right (HR), Fore Right (FR)—see [4])—equivalent to Aristotle's description. However, this stability account is incomplete, as walking quadrupeds fail to maintain more than two feet on the ground at all but the very slowest speeds (highlighted by Gray [2]; and even at the slowest speeds, instants of two-foot support can often be observed in horses); without at least a tripod of support, static stability is not continuously maintained. While an energetic account has recently been proposed [5]—for quadrupedal walking at moderate speeds—that successfully explains the different phasing between different animal groups (‘normal’ and ‘slow or slow-muscled’), this approach must also be incomplete. Firstly, it relies on the assumption that the limb force profiles through time are broadly invariant. This is incorrect for very slow gaits—during grazing, for instance—where the timing of steps can vary considerably, and the demands of at least some postures allowing static stability are unavoidable. Secondly, while this approach finds that the diagonal sequence footfall pattern (HL, FR, HR, FL) of primates [6] achieves a local minimum in terms of mechanical work, it is not the global minimum as found for the ‘normal’ lateral sequence of horses. Our motivation is therefore to account for not only the footfall order but also the footfall timing of the gait of grazing animals, and to extend this model to provide a novel, putative account for the walking gait of primates and some other branch-walkers [7]. The diagonal sequence (we model here a phase of 75%) footfall pattern of primates (usually observed to be somewhat less than 75%) requires explanation: it deviates further from both serial static stability (see electronic supplementary material, movie S2) and mechanical work minimization [5] than the ‘normal’ horse-like phase of 25%. A current—perhaps prevailing—view (though many exist—see [8] for history) is that the primate phasing allows greater stability at the instant of forefoot placement (see [9]). However, the mechanism underlying this possibility is unclear: if continuous static stability is possible with a lateral (horse-like) sequence, but not with a diagonal (primate) sequence (contrast electronic supplementary material, movies S1 and S2), how might the diagonal sequence reduce rolling or pitching at forefoot placement?

## 2. The grazing gait

**Figure 1.**
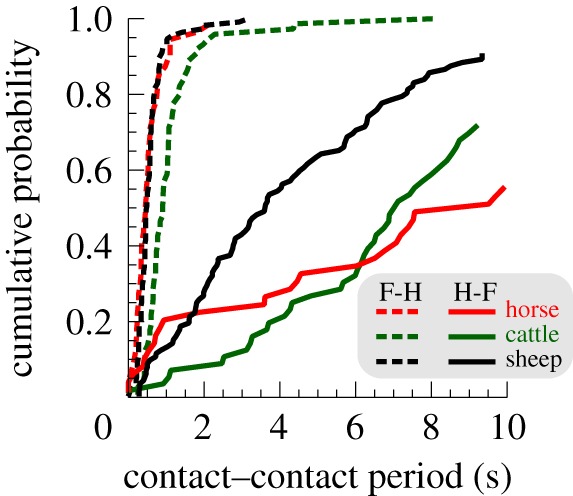
Intervals between hind and fore (H-F), and fore and hind (F-H) foot placements during grazing. A step forwards with a forefoot is directly followed by a step forwards with the hindfoot; the opposite is not true.

The gait during grazing is not conventionally included in any list of discrete gaits, perhaps because the timing between steps can be highly variable, the motion is intermittent with instants of near-zero forward motion, or because the footfall order is the same as for walking. However, while a typical walk has a duty factor around 0.65 (each foot spends 65% of the stride cycle on the ground) and HL–FL phase of 25% (of the HL–HL stride period), grazing involves a duty factor approaching 1 and phase close to (but importantly not) 50%. We therefore introduce the concept of progression during grazing as a gait (see electronic supplementary material for further discussion). Footfall timings taken from videos of grazing (head down, generally forward motion) sheep, horses and cattle show that static stability is achieved at some point (probably many points) before forefoot motion: the hind-on to fore-on duration can be very large (many tens of seconds) and variable. In contrast, movement of the front foot is consistently and tightly followed by repositioning of the rear foot (table 1, figure 1).

**Table 1.** Summary statistics of fore–hind and hind–fore foot placement intervals for a range of grazers.

	fore–hind			hind–fore		
	sheep	horses	cattle	sheep	horses	cattle
*N* (individuals)	8	6	11	8	6	10
mean of individual median contact–contact period (s)	0.44	0.35	0.98	5.00	14.90	8.44
s.d. of individual median contact–contact period (s)	0.12	0.15	0.32	2.77	10.03	3.81
coefficient of variation	0.27	0.43	0.33	0.55	0.67	0.45
sign test of null hypothesis that median fore–hind interval is equal to median hind–fore						
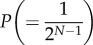	sheep	horse	cattle			
	0.008	0.031	0.002			

## 3. The toppling table model—general

In order to address the observation of footfall timings in grazers and walking primates, we develop here a simple model to consider the geometric implications of different hind–fore phases assuming that each hip and shoulder rises and falls over a stance, and that each hip and shoulder is linked by a rigid back that resists twisting and bending. These simplifications are clearly extreme, and demonstrably wrong in detail (see electronic supplementary material for further discussion); however, they allow calculation of limb and back geometries that offer general insight.

*Assumption 1*. Each hip and shoulder goes from low to high to low over each stance. *Justification 1*. Leg length is approximately constant through stance in bipedal (human) walking gaits, presumably to achieve work-minimizing, vaulting stances. At very low speeds mechanical work and power demands *per se* are probably negligible. In this case, constant leg length may offer benefits due to maintaining a conserved optimal limb posture that minimizes costs of static weight support. By analogy with human legs: while we are able to stand still supporting our weight with flexed knees, or while standing on tiptoes, our normal standing posture is less tiring owing to alignment of ground reaction forces broadly through joint centres, and reduced demand for muscle activation. *Implication 1*: The height of each corner of the back—linked to hip or shoulder—rises then becomes lower over the period of the relevant leg's stance.

*Assumption 2*. A rigid, table-top-like connection between each hip and shoulder. *Justification 2*. Some form of linkage between the legs during normal gait is obvious: the phase of left and right leg action during locomotion is not random—in walking and running bipedal gaits it is consistently very close to 50%. Further, some linkage is apparent between hind- and forelimbs in quadrupedal gaits. While these linkages may be neural [8], and to some extent genetically or developmentally determined, they may also (and not exclusively) be attributed to simple mechanical connections. Here, we consider some of the implications of a mechanical linkage between hips and shoulders, specifically assuming resistance in terms of bending and twisting: the back is treated with the extreme case as acting as a totally rigid table-top with hips and shoulders defining the corners. *Implication 2*: This assumption allows the coordinates of the back to be fully determined given the positions of three of the four hips and shoulders. Only under specific conditions can all four legs be loaded (analogous to the wobbling of a four-legged versus three-legged stool).

**Figure 2.**
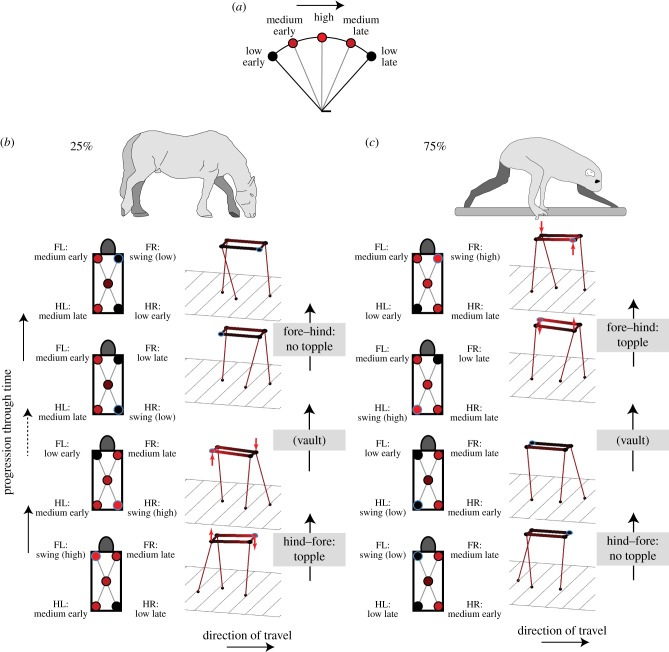
Changes in height due to stiff-limbed vaulting over stance (black low, red high) for a single hip or shoulder (*a*) or all hips and shoulders for instants of hind–fore and fore–hind foot transitions for ‘normal’ horse-like (*b*) or typical ‘primate’ walking (*c*) foot sequencing. The height of three corners of the table-top-back are defined by the height of the hips and shoulders connected to legs in stance; the fourth corner (blue circle outline) is defined by the plane of the back. At the instant of one of the foot placements, there is a discontinuity in back orientation, requiring rapid pitching and rolling (red arrows). This instant occurs at forefoot placement in ‘normal’ walking, but hindfoot placement with the ‘primate’ sequence. The primate sequence may promote careful, controlled forefoot placement suitable for walking along branches. Horse and loris [10] show geometry at the instant of forefoot placement.

Using Assumptions 1 and 2, we calculate the height surface of a table-top-like back throughout a gait cycle using a duty factor of 0.75 and 25% (normal, horse-like lateral sequence walking; electronic supplementary material, movies S1 and S1b) or 75% (diagonal sequence, primate) phase (electronic supplementary material, movies S2 and S2b). With these parameters, all the heights of all points on the ‘back’ are explicitly defined as, at all instants, the heights of three corners of the table-top (two shoulders and one hip, or two hips and one shoulder) are provided by the state of each of the exactly three supporting legs (figure 2). We find that only at one instant in a gait cycle can four legs be loaded; and at another instant the back must topple—rolling and pitching quickly between one position and another (figure 2*b*,*c*). *We view this geometrical insight as the major advance of the paper.* The discontinuity in back orientation is not dependent on centre of mass positioning or fore–hind weight bias. It relates to a quite different mechanism from that proposed previously [9], which focuses on deviations from static stability (i.e. centre of mass over the polygon of support) at duty factors less than 0.75 (and need not apply at duty factor ≥ 0.75)

In order to consider grazing, we assume that when duty factor > 0.75, there are periods during which one of the limbs may be in contact with the ground, but need not be loaded.

## 4. The toppling table model applied to grazing gaits

Grazing clearly achieves long periods of ‘serial static stability’—many instants through a gait cycle in which motion can be absolutely stopped. This can be achieved with a phase of 25% (and not 75%) at the lowest duty factor—0.75 (see electronic supplementary material, movies S1 and S2). However, this is not a sufficient requirement to account for footfall timing as duty factor approaches 1.

### (a) Protraction assumption *straw man*: stability margin is maximized

This might be the default hypothesis given the requirements for static stability. Is a footfall timing used that maximizes the distance between the centre of mass location and any edge of the polygon of support formed by the feet? No: this would be achieved by moving the forefoot forwards immediately after motion of the hindfoot, the feet forming parallelograms of support (electronic supplementary material, movie S3; movies S3 and S4 are also presented as 3D Blender animations). Instead, the opposite is observed, with hindfoot moving directly after forefoot, forming isosceles trapeziums of support.

### (b) Protraction assumption: swing limb forwards when maximally unloaded

This would minimize any disruption to weight support, and additionally allow protraction with minimal requirement for bending the leg to avoid ‘scuffing’ the foot along the floor (electronic supplementary material, movie S4). This assumption *is* consistent with the foot timing used during grazing: as the animal moves forwards the forelimb becomes increasingly unloaded due to the rolling and pitching geometry of the table-top-back. At the extreme of stance, the forelimb is maximally unloaded; it then swings forwards. According to the model geometry, the table-top-back immediately topples onto the newly placed forelimb, lifting the diagonal hip and maximally unloading the supporting hindlimb, which is observed to swing forwards almost immediately rather than ‘left hanging’ (electronic supplementary material, movies S5 and S6).

## 5. The toppling table model contrasting ‘normal’ and ‘primate’ walking patterns

The toppling table-top-back geometry indicates that, with a ‘normal’ horse-like footfall phasing, there is a moment when—if left uncompensated (which presumably has energetic or computational costs)—there is an instant of rapid rolling and pitching at the instant of forefoot placement. Whereas this presents little problem during walking on a surface, as a mistiming of foot placement will not result in a misstep, it may be unfavourable when walking along a branch, which requires accurate positioning and timing of foot placement. The diagonal (typical primate) limb phasing, in contrast, results in the instant of forefoot placement coinciding with the instant at which all four limbs can be loaded without toppling—that rare condition where a four-legged stool truly stands on all four legs without rocking. The moment of rapid rolling and pitching instead occurs during hindfoot placement, which may be less challenging as the substrate has already been tested, and close hind-on-previous-fore placement would allow proprioception rather than vision to guide placement onto the branch. We therefore support the concept [9] that the primate footfall sequence may be driven by issues of forefoot placement on a narrow or uncertain support, but tentatively propose a contrasting mechanism.

## 6. Conclusion

This extreme, simplifying geometric reduction of a stiff table-top-back linking hips and shoulders that rise and fall over each stance reveals periods of toppling, even in gaits with very high duty factors, and allows accounts for footfall timing (grazing gaits) and sequencing (horse versus primate) that cannot currently be attributed purely to energetics or static stability.

## Supplementary Material

Supplementary Discussion.docx

## Supplementary Material

Movie S1.mov: animation of DF=0.75, phase=25% walking model

## Supplementary Material

Movie S1b.mov: animation of DF=0.75, phase=25% walking model, with annotations

## Supplementary Material

Movie S2.mov: animation of DF=0.75, phase=75% walking model

## Supplementary Material

Movie S2b.mov: animation of DF=0.75, phase=75% walking model, with annotations

## Supplementary Material

Movie S3.mov: animation of an incorrect model of the grazing gait

## Supplementary Material

Blender movie S3.html: animation of an incorrect model of the grazing gait.

## Supplementary Material

Movie S4.mov: animation of the good model of the grazing gait

## Supplementary Material

Blender movie S4.html: Animation of the good model of the grazing gait

## Supplementary Material

Movie S5.mp4: Typical side view video of a grazing quadruped

## Supplementary Material

Movie S6.mp4: Grazing horse video with variable playback speeds

## Supplementary Material

Frame numbers.xlsx: Spreadsheet of frame numbers

## Supplementary Material

ExcelSurfaceCode.xlsx: An Excel workbook that calculates back height surfaces

## Data accessibility

Legends for the supplementary material, a spreadsheet containing the frame numbers processed to create figure 1 and table 1, and an Excel workbook which calculates back height profiles, have been uploaded as part of the electronic supplementary material.
