# Supplementary material for: The grazing gait, and implications of toppling table geometry for primate footfall sequences
Source: Biol Lett. 2018 May 16;14(5):20180137. doi: 10.1098/rsbl.2018.0137 (PMC6012707; doi:10.1098/rsbl.2018.0137)
Supplement: Supplementary Discussion.docx [file rsbl20180137supp1.docx]

*Supplementary Discussion 1: A brief discussion of gait definitions*

Of interest here is what defines a ‘gait’, and what makes it sufficiently different from some other ‘gait’ to justify its own designation. This depends somewhat on the context of importance to the author. We focus on two of the most widely used definitions, at least within the field of comparative biomechanics.

Hildebrand (1989)^1^ states ‘*Each of these animals illustrates a different gait, or regularly repeating manner of moving the feet*.’ This suggests that, for Hildebrand, regularity is an important characteristic of a gait. In some part, this may be attributed to the techniques pioneered by Hildebrand in describing gaits in terms of duty factors and various phases; these require there to be some regularity through time for a repeating gait cycle. However, this definition (while appropriate for Hildebrand’s purposes at the time) is by no means universal. Several pathological gaits are characterized by departure from regularity: the Parkinsonian gait ‘may show an involuntary inclination to take accelerating steps…’ and in the Choreiform (hyperkinetic) gait, ‘the patient will display irregular, jerky, involuntary movements in all extremities’^2^.

Therefore, while helpful when describing many of the gaits of quadrupeds, regularity should not be considered an outright requirement for a gait.

An alternative definition is provided by Alexander (1989)^3^.

*‘In this paper, I adopt the following definition of “a gait,” which I believe to be consistent with current usage.*

*A gait is a pattern of locomotion characteristic of a limited range of speeds described by quantities of which one or more change discontinuously at transitions to other gaits…’.*

This gait definition suggests that there should be something discrete about a transition from one gait to another. While this approach has value when considering the different mechanical principles that might be dominating under different conditions – a laudable goal and the one being pursued by Alexander in 1989 – a couple of examples demonstrate limitations with this definition. While the walk-run transition in adult humans fits Alexander’s definition beautifully, showing discontinuities in a number of quantities, the walk-run transitions of small birds^4^, young humans^5^ and elephants^6^ are far from discrete, and declaration of a relevant discontinuity would appear arbitrary.

However, from a biomechanical perspective, identifying and even defining a ‘gait’ in terms of its underlying mechanics remains appealing. In this context, the concept of a ‘grazing gait’ appears valid and has utility. It is easy to distinguish this gait from the typical walking gait of livestock even though the transition has not been described: the grazing gait has a duty factor approaching 1 instead of around 0.65; a phase approaching (but below) 0.5 instead of 0.25; and variability in hind-fore contact timing but not fore-hind. It can also be attributed to fundamentally different mechanical demands – economy of serial static support and the toppling-table principle (this paper) vs. mechanical economy of locomotion.^7^

1.Hildebrand M (1989). The quadrupedal gaits of vertebrates. *Bioscience* 39, 766-775.

2. https://stanfordmedicine25.stanford.edu/the25/gait.html

3. Alexander R McN (1989). Optimization and gaits in the locomotion of vertebrates. *Physiological Reviews*, 69, 1199-1227.

4. Gatesy SM and Biewener AA (1991). Bipedal locomotion: effects of speed, size and limb posture in birds and humans. *J. Zool*. 224, 127-147.

5. Hubel TY and Usherwood JR (2015). Children and adults minimize activated muscle volume by selecting gait parameters that balance gross mechanical power and work demands. *J. Exp. Biol.* 218, 2830-2839.

6. Hutchinson JR, Schwerda D, Famini DJ, Dale RHI, Fischer MS and Kram, R (2006). The locomotor kinematics of Asian and African elephants: changes with speed and size. *J. Exp. Biol.*, 209, 3812-3827.

7. Usherwood, JR and Self Davies ZT (2017). Work minimization accounts for footfall phasing in slow quadrupedal gaits. *eLife* 2017;6:e29495 doi:10.7554/eLife.29495.

*Supplementary Discussion 2: Validity of model assumptions for primates versus livestock.*

The assumptions of the table model are less well met with most primates, which flex their limbs considerably during stance^1^, potentially related to issues of scale ^2,3^ and/or avoidance of branch-damaging loading^4^. Nevertheless, Assumption 1, that *each hip and shoulder goes from low-to-high-to-low over each stance* does appear to have some empirical support, at least for the forelimbs of catarrhine primates walking on terrestrial and arboreal supports^5^.

Bending and twisting of the back may be more pronounced among climbers^6^ than for livestock, presumably making Assumption 2, of *a rigid, table-top-like connection between each hip and shoulder* less valid. However, very similar concerns can be raised with the currently prevailing concepts relating footfall patterns to models of static stability, in which the center of mass is assumed to maintain a fixed position in the horizontal plane with respect to hips and shoulders, and periods without tripod support are – while assumed to be in some way deleterious – still permissible. That an achievable degree of rolling can circumvent the stability issues is easily demonstrated with personal experience in adult human crawling. It is perfectly possible to maintain quadrupedal crawling on hands and knees using all sorts of hind-fore phases, including a pure ‘pacing’ phase, with periods of only left knee and left hand supporting the body. In part, this is due to the dynamic nature of crawling; but also slow crawling is possible with this support pattern due to a small degree of tipping (rolling) of the body and legs, allowing the center of mass to be placed sequentially over the left pair then right pair of supports. Further, conventional stability concepts have difficulty with supporting a body walking along a narrow branch, whereas the principles behind the table model still apply. These points are made in order to highlight that the current model, while admittedly resorting to extreme assumptions, at least makes these assumptions explicit; and that previous models providing the current paradigm are not free from similar assumptions – though they are rarely expressed explicitly.

We acknowledge that the table model can only consider a duty factor of exactly 0.75 and phases of exactly either 25% or 75% (the only conditions for which three, and only three, points of the back surface are continuously defined), and that these conditions do not precisely match typical horse or primate walking. However we point to the insight provided by extreme reductionist models of bipedal walking models: these are often limited to an unrealistic duty factor (exactly 0.5), but have done great service in explaining basic aspects of adult human walking gaits.

*References*

1. Larney E, and Larson S.G. (2004). Compliant walking in primates: elbow and knee yield in primates compared to other mammals. *Am J Phys Anthropol* 125:42-50.

2. Biewener AA (1989). Scaling body support in mammals: limb posture and muscle mechanics. *Science* 245, 45–48.

3. Usherwood JR (2013). Constraints on muscle performance provide a novel explanation for the scaling of posture in terrestrial animals. *Biology Letters* 9.

4. Schmitt D, Cartmill M, Griffin TM, Hanna JB, and Lemelin P. (2006). Adaptive value of ambling gaits in primates and other mammals. *J. Exp. Biol.* 209:2042-2049.

5. Schmitt, D. (1999). Compliant walking in primates. *J. Zool. Lond.* 248, 149-160.

6. Shapiro L.J., Demes B, and Cooper J. (2001). Lateral bending of the lumbar spine during quadrupedalism in strepsirhines. *J. Hum. Evol.* 40(3):231-259.
